# Grieving the Living: The Social Death of Former Jehovah’s Witnesses

**DOI:** 10.1007/s10943-020-01156-8

**Published:** 2021-01-19

**Authors:** Heather J. Ransom, Rebecca L. Monk, Derek Heim

**Affiliations:** Department of Psychology, Edge Hill University, Ormskirk, L39 4QP UK

**Keywords:** Jehovah’s witnesses, Ostracism, Identity, Self-esteem, Mental well-being

## Abstract

Addressing a relative lack of research investigating the experiences of individuals who have left the Jehovah’s Witnesses (JW), this research utilizes a social identity approach to examine qualitatively, the process of transitioning towards post-JW life, experiences of ostracism and perceived threats to self-identity. Semi-structured interviews were carried out in the homes of six former JWs, and transcripts were analysed using interpretive phenomenological analysis. Narratives suggest that experiences of ostracism following religious exit can be associated with diminished mental health, while having a sense of agency and establishing new (online) social connections may help mitigate adverse consequences. Implications and future research directions are discussed.

## Introduction

Founded in 1879 by Charles Taze Russell, Jehovah’s Witnesses (JWs) are a “world rejecting” (Wallis 1984) fundamentalist Christian group which has as a central belief that Armageddon is imminent and will allow “God’s kingdom” to be established (Stark and Iannaccone 1997). This religion maintains that all wickedness on earth will be destroyed during this cataclysmic event and that its followers will survive as part of “the great crowd” to enjoy a thousand years of paradise on earth (Holden 2002). With a worldwide membership of over eight million, JWs are active in 87 countries and members cumulatively spend more than two billion hours annually preaching their message. JWs do not report disaffiliation figures, and although previous research indicates that they had sustained growth of about 5% per year for the previous 75 years (Stark and Iannaccone 1997), more recent figures from their website (jw.org) suggest this growth slowed to 1.3% between 2018 and 2019 (Jehovah’s Witnesses 2018, 2019). Research from the 2014 Religious Landscape Study involving over 35,000 Americans (Pew Centre 2018) indicates that the group has among the lowest retention rate of any religious tradition with 66% of those raised JWs no longer identifying with the group, and that 65% of all adult JWs converted into the faith. While it is difficult to ascertain verifiable statistics, this work implies that many people appear to leave the faith.

The JWs strict moral code may be implicated in religious exits. Members are required to act in accordance with prohibitions that include sex outside marriage, masturbation, pornography, homosexuality, gambling, drug and tobacco use (Stark and Iannaccone 1997). Baptised members risk being disfellowshipped when transgressing these requirements which, following an interrogation by a “judicial committee” of three “elders” (church leaders) to ascertain the gravity of a rule contravention, can entail ejection from the faith (Stein 2012). JWs may also depart by disassociation, whereby an individual officially “resigns” from the faith, usually in writing. Disassociation can also be imposed on an individual who voices disagreement with doctrine, or if church elders hear of, or witness, contraventions (e.g. celebrating Christmas). In this way, a JW is said to “disassociate themselves by their conduct” and this also results in immediate shunning by the JW community. Third, a JW may leave by “fading”, whereby an individual quietly ceases worship (e.g. attendance at JW services) and activities such as public preaching. In the past, fading was not met with mandated ostracism, making it a preferred exit method; however, recent research indicates that former JWs who exited in this way also report experiences of social “shunning” (Ransom et al. 2020).

While JW followers benefit from a ready-made social network, along with the prestige that comes with membership, JWs are considered a “high-cost” group (Scheitle and Adamczyk 2010), as exiting can have negative consequences, including loss of supportive ties, challenges to self-perceptions and psychological distress (Berger 2015; Fazzino 2014; Fenelon and Danielsen 2016; Iannaccone 1994). Indeed, those who exit the JW faith often face the prospect of severing significant relationships with family and friends (Miller 1988), which can be associated with feelings of loneliness and abandonment (Testoni et al. 2019) and pose challenges to health and well-being (Friedson 2015). However, while experiences of ostracism are commonplace amongst former JWs, the effects of this, including potential negative impacts on well-being and mental health remain under-researched (Knox 2017).

Regardless of whether leaving the JWs was voluntary or not, former members often report being ostracised and rejected by the religious community they had previously been a part of (Holden 2002; Ransom et al. 2020). The wider psychological literature provides evidence with regard to how this can impact individuals adversely (Case and Williams 2004; Williams 2001, 2008). This is illustrated by Williams’ (2009) needs threat model according to which ostracism proceeds in three stages: first, a reflexive stage whereby the target experiences threats to one or more fundamental needs (self-esteem, belonging, control, meaningful existence), second, a reflective stage where the target reflects on how to repair perceived threats and finally, a resignation phase, whereby coping resources can become depleted as repeated efforts to re-establish relationships fail.

While this framework has yet to be applied in the context of JWs, evidence converges to suggest that examination of the effects of perceived ostracism among those leaving high control religions is warranted. This is because studies from other faiths point to the detrimental effects on identity and well-being that can occur when leaving strict religious environments (Scheitle and Adamczyk 2010). Indeed, leaving religiously exclusive groups has been suggested to be associated with diminished health and well-being, while anticipated ostracism and identity loss have been identified as significant barriers to exit (Scheitle and Adamczyk 2010). Likewise, accounts from those who have left the Jewish Ultra-orthodox (Berger 2015) and Mormon faiths highlight difficulties in merging incompatible identities (Joseph et al. 2017). These studies in other faiths therefore reveal an emerging theme of threatened self-identity resulting from ostracism post-leaving and highlight this as a possible area of investigation among JWs leavers.

The formative work by Hookway and Habibis (2015), who undertook an in-depth exploration of leaving narratives of former JWs, illustrates this further. Here, respondents describe transitioning out of the JWs as a painful process due to the perception that their JW faith is incompatible with their new chosen lifestyle and values. Similarly, Lalich and McLaren (2010) document accounts of suicidal ideation and/or self-destructive behaviours amongst gay/lesbian former JWs which, they believed, were caused by difficulties in merging their strictly defined JW identity with their homosexual identity. This is echoed by findings from a recent study in which former JWs describe the consequences of disaffiliation from the faith as being akin to a mourning process due, in part, to a perceived loss of identity and social ties (Testoni et al. 2019). Testoni and colleagues’ (2020) more recent qualitative study examining the loss and grief that occurs when people’s loved ones go missing usefully highlights possible commonalities with regard to the barriers that affect individuals who seek some form of closure (Testoni et al. 2020). Here, in accounts that appear to show similarities with those of JWs, distressed relatives of those who are missing in Italy report an ambiguous form of loss, anticipatory mourning and grief, with an uncertainty of resolution (c.f., Hookway and Habibis 2015; Lalich and McLaren 2010; Testoni et al. 2019). Existing studies have therefore begun to paint a picture of the experiences of former JWs, and wider literature on ostracism and complicated grief processes appear to provide useful insights into how potentially negative outcomes associated with leaving the JWs may unfold.

There is by now also an ample body of theory-driven evidence highlighting both advantages of religious affiliation as well as possible drawbacks of exiting religious faiths (Baker et al. 2018). For example, benefits that religious membership can bestow on self-esteem through the belief in a secure relationship with the “divine” (Aydin et al. 2010), social support (Gebauer et al. 2012), instilling feelings of commonality (Pargament et al. 2000) have been made apparent. Considering the significant role of social ties in these studies, it is possible that the benefits of religious membership may, in part, be explained by the well-established tenets of the social identity approach (Brown 2020: Tajfel 1978; Tajfel and Turner, 1979). This perspective suggests that membership of social groups can help create a meaningful sense of belonging (Gomez et al. 2011; Turner et al. 1987; Williams 2001), and alignment with groups can result in a visceral sense of oneness with the group, or a *fused* identity (Gomez et al. 2011). Conversely, wider social psychological literature indicates that detachment can be associated with negative effects to well-being and one’s sense of belonging (Baumeister and Leary 1995). Leaving a social group therefore can pose threats to people’s sense of identity and which may require the initiation of a process towards constructing a new identity (Breakwell 1986, 1992; Jaspal and Cinnirella 2010).

Applying a theoretical lens relating to identity reformulation, commonalities may therefore be drawn between leaving high control religions and desistance from crime and substance use. Here, researchers describe a process of “transitioning out” of former groups and postulate that providing individuals with new supportive social groups may increase likelihood of recovery and decrease incidences of relapse (Best et al. 2016; Best et al. 2017; Buckingham et al. 2013; Beckwith et al. 2019; Frings and Albery 2014; Haslam et al. 2019). Although transitioning away from a religious group is not the same experience as portrayed in the desistance from crime and substance use literature, commonalities can be identified. For example, both groups typically face social losses and, to a greater or lesser extent, need to “reinvent” themselves regarding how they position themselves vis-à-vis different social groups. However, differences are also noted in that those seeking a path away from addiction or crime may forge a new social identity through choice, whereas this may be a necessity for exJWs. Further, those transitioning their identity away from harmful addictions frequently receive family support in this endeavour, whereas exJWs typically lose family relationships through mandated ostracism. At the same time, the proliferation of online support groups for both former addicts and exJWs may be partly explained by how these forums facilitate identity transition (Avance 2013; Best et al. 2018; Scharp and Beck 2017).

With the above in mind and the purpose of contributing to the literature regarding the effects of leaving high-cost religions, the aim of the current research is therefore to analyse qualitatively the experiences of those who have left the JWs. Specifically, the study examines how people negotiate the process of transitioning towards post-JW life and the extent to which experiences of ostracism and threats to identity may impact on this process.

## Methodology

### Participants

Six former JWs were recruited using purposeful sampling by contacting acquaintances (*n *= 2) of the interviewer and seeking participants via Facebook through posts to relevant forums (*n *= 4). Participants were British and aged between 25 and 65 years. Two were baptised into the faith as adults and four as teenagers. All participants were currently experiencing religious shunning from family and friends within their former respective JW communities. Table 1 uses pseudonyms and summarises (i) participant characteristics, (ii) reasons for leaving, (iii) the exit process and (iv) details of current ties with friends and family who remain JW members.

**Table 1:** Respondents’ characteristics and exit stories at the time of interviews

Respondent characteristics	Reasons for leaving	Exit process	Current ties with JW friends/family
John (PP1), 25-year-old white male born into the JW faith. Inactive for four years. Social support after leaving	John left the JWs through choice because he felt that he could not live the life he wanted within the JW faith. He felt the organisation was overly strict, strongly discouraging further education and limiting life choices, commenting that “*it’s not what I believe worship to God should be like*”. He describes his previous faith as: “*a mesh of conformity and self*-*regulation*” that he would never return to	John decided to stop attending religious services and fade out of the faith together with his mother and brother. He felt that having support eased the transition out of the faith commenting that “*we grew stronger…supported each other personally rather than through a construct of religion”*	John lost a significant number of family relationships which included with his sister, father, grandparents, uncles and aunts, and cousins he had previously been close to. He surmised that they *“…that did not think we made the right decision; they keep their distance from us now”.* John felt that relationships with all other family members, including his mother and brothers remained intact, and felt that his relationship with these individuals had improved because of leaving together, commenting *“we got to know each other better”*
Sue (PP2), 51-year-old white female born into the JW faith. Disfellowshipped aged 25 years with no social support, and reinstated age 26 years, then disassociated (resigned) 28 years ago	Sue was disfellowshipped from the JWs against her will a few days after her wedding, due to JW leadership viewing her marriage as “adulterous”. She said that this was because her new husband had not been divorced on “scriptural grounds” according to JW doctrine and was therefore not “free to marry”, constituting her as an “adulteress”. Two years after reinstatement into the JWs, Sue disassociated herself from the JWs along with her husband (28 years ago), because she no longer believed the doctrine	Sue found her first exit from the faith very difficult because she still believed the doctrine when she was disfellowshipped. Commenting on her level of devotion she said: *“I was 101% JW, didn’t question anything, believed it 100%”.* The second time she exited, it was through choice. She comments *“because I had started attending a church, they were trying to disfellowship me. So, it was either jump or be pushed, so in some ways it wasn’t our choice, but I didn’t want them to do that* (disfellowship) *to me again”*	Family relationships including parents, both sisters, all nephews and nieces were all lost. Sue comments *“all my family and friends were still in it (JWs), so the rejection, how they felt about me, how they treated me, nothing changed”* Sue compared the loss of all of her friends and family to a plane crash, commenting: *“Overnight, it was like everybody that I knew in my life had got on a jumbo jet and it had gone down over the Atlantic … but there was no closure because everyone was still living their lives”*
Jo (PP3), 72-year-old white female converted at age 27 years by JWs who came knocking on her door. Left the JWs voluntarily in 1993, (with social support) age 47 years as she no longer believed the doctrine and felt there was no love there	Jo left the JWs 24 years ago for two reasons. First, she decided there was no love in the congregation, describing church elders as “*three demons, like bulldogs … seething with anger”*. Second, having become convinced that the world would end in 1975: *“Armageddon was coming in 1975, me and the children were going to be saved”.* She became suspicious when this did not happen and finally walked out 18 years later, never to return to the JWs	Jo decided to stop attending religious services and to fade out of the faith. Because she no longer believed the doctrine and did not have many JW friends, exit was not challenging. She comments *“I felt very lonely…I met Mark that night, as they say…one door closes, another door opens…I was on my own but determined to make friends”*	In 1993 when Jo left the JWs, she did not experience shunning until 2011. She recollects the night when her family (her son, his wife, and their three children) failed to arrive for a meal: *“I’d cooked the dinner and they hadn’t even told me…that was the start of it…I tried every day after that”.* Still being shunned at the time of interview she comments *“I haven’t seen him in six years, he’s not my son”.* Jo still has a good relationship with her daughters who left the JWs with her in 1993
Sophie (PP4), 38-year-old white female, born and raised in the Jewish faith, converted to the JWs at university, aged 20 years. Disfellowshipped twice, finally exiting the faith in 2013	Sophie did not want to leave the JWs but was disfellowshipped twice. The first time (1998) for having sex before marriage, and the second time (2011) for exposing domestic violence. Sophie finally quit trying (and failing) to get reinstated five times before 2013, after researching the JW religion. Since then she found support in the ex-JW Facebook community, commenting *“I am mixing with a lot of ex*-*JWs and other cult survivors…using my critical thinking skills to break it down”*	Sophie did not want to leave (disfellowship) the JWs and found the exit process very challenging. Resulting in hospitalisation with “reactive psychosis” as a result of perceived unjust treatment, she said of her situation*: “I was disfellowshipped for exposing domestic violence, so it felt very unjust”.* She had been told by the church leaders not to report the domestic violence to the police as *“exposing domestic violence goes against what Watchtower want to present themselves as”*	Although both of her children were attending JW meetings when she was disfellowshipped, her 18-year-old daughter refused to comply with shunning her mother and also left the JWs. Sophie lost custody of her ten-year old son for five years saying that *“it caused a lot of mental instability… my JW husband took custody of my son… after he abused my daughter”.* At the time of interview both her children remained outside the religion. Sophies parents are Jewish, so her relationships with them remained strong. She lost all of her JW friends
Claire (PP5) in her sixties, white female, who was raised a JW from infancy. Disfellowshipped twice with no social support. Left the JWs 30 years ago	Claire was disfellowshipped twice from the JWs, first when she was 15 years of age, and a second time at age 30 years, after fleeing an abusive marriage. After her second reinstatement she left the JWs voluntarily deciding she no longer believed the doctrine	Claire found exiting from the JWs difficult. As an isolated 15 year old she recalls *“it was very difficult, all your friends, all the people you know are JWs, so the only way back is to go back, which involved sitting at the back of the hall being ignored by everybody”.* Commenting on the second time it happened she said *“when you’re disfellowshipped, you are made to feel repugnant, like a dog returning to its vomit…you feel a lesser person, stripped of everything and all the friends you had…you’re a nothing…and a nobody”.* Although leaving 32 years ago, only in recent years has she found limited “recovery” through Facebook groups for ex-JWs, commenting *“I can’t express enough really how much that has helped me”*	At the time of interview, Claire was being shunned by her (JW) eldest son, commenting *“he lives literally ten minutes away. I don’t think I’ve seen him for nine months”*. She talks about her JW daughter who permits very limited contact. *“her comments to me are very brief and she’ll make an excuse she has to go”.* From her second marriage Claire had a son who committed suicide after being disfellowshipped from the JWs. She also has two other children with whom she maintains good relationships
Joanne (PP6), 48-year-old female. Raised from infancy in the JWs. Disfellowshipped with no sources of support	When she was disfellowshipped in 2013 for sex outside of marriage, Joanne was a firm believer in JW doctrine, and had originally intended to be reinstated into the faith. During the time of her interview, however, she expressed a hatred for the JW religion, due to the shunning she had experienced. She exclaimed that now she had overcome her fear of “Armageddon”, she has no desire to return to the JWs, commenting *“you live your whole life in fear of Armageddon…that you’re going to die”*	Joanne found the exit process challenging as she remained a believer when she was disfellowshipped in 2013 commenting *“I really strongly believed in it and never stopped believing in it until recent years”.* Describing her former self as highly sociable, she found losing relationships especially challenging. As an only child, she particularly grieves the loss of her mother, stating: *“if your own mother can turn around and do that, then I have no faith in people…I have nothing…I have lost everything”*	When she was disfellowshipped, Joanne lost relationships with two of her three children, who wanted to remain JWs, and felt compelled to comply with mandatory shunning. Joanne’s son was disfellowshipped shortly after her, and their relationship had been restored. Her mother and daughter, at the time of the interview, were still shunning her. Her youngest son was seven when she left the JWs and she described their relationship as strong

## Materials

All participants were interviewed using the same in-depth semi-structured interview schedule. To facilitate a flexible inquiry, interviews started with open ended questions, with prompts/probes to elicit in-depth accounts. Questions such as “Can you tell me how you came to leave the Jehovah’s Witness religion” were designed to explore the experience of leaving the JW faith. The interview schedule was designed to explore effects to individuals’ sense of self, self-esteem, and feelings of belonging after leaving the JWs. Respondents were invited to elaborate on these issues through open-ended prompts such as “Can you tell me a little more about that”, in order to obtain rich accounts. These accounts were then transcribed for analysis of detailed verbatim transcripts.

## Procedure

Interviews were carried out by a former born-in JW who left the organisation voluntarily five years prior to conducting the interviews. While it is likely that the interactions with respondents was coloured by this (see Merton 1972), as a former JW and therefore an “insider researcher”, it was possible to collect naturalistic data, and utilise unique insights and perspectives that others not acquainted with the JW faith may not have been able to contribute (Trowler 2011). Specifically, the researcher had the ability to understand in possibly a nuanced fashion the procedures and tenets of the JW faith described by participants, which may have added cultural literacy to the research (ibid).

Interviews lasted approximately 1 h and took place in participants’ own homes so they could feel comfortable telling their stories of exit, guided by the semi-structured interview schedule. Ethical approval from Edge Hill University ethics panel was obtained prior to commencing this research. As discussions of shunning can be painful, care was taken with each participant to inform them, that at any time the interview could be stopped. Some participants exhibited distress on occasions as they recalled emotional events from the past, especially when discussing the experience of being disfellowshipped. When these respondents were reminded that the interview could be stopped at any time, this was refused on every occasion. On the contrary, respondents appeared determined to “tell their stories”, and afterwards described the experience as cathartic. One respondent expressed that she felt a healing effect from the interview, remarking that the interview was the first time she had revealed her story.

## Analysis

Interview transcripts were analysed using interpretive phenomenological analysis (IPA), an idiographic method chosen because it relates to how individuals make sense of, and give meaning to, major life events of personal significance (Smith et al. 2008) from their own standpoint (Patton 2002; Smith et al. 2008). Specifically, IPA was deemed appropriate for the present research as it aimed to understand what a given experience of ostracism feels like (phenomenology), and how individuals make sense of it (interpretation). Having origins in hermeneutics (meaning-making) and phenomenology (the study of phenomena or experience) IPA combines psychological, interpretive and idiographic processes to gain insights into the experiences of each individual, in their given context, to make sense of a given or shared phenomenon (Gill 2014). IPA’s hermeneutic stance is one of inquiry and meaning-making (Larkin et al. 2006), and so the researcher seeks to make sense of the participants’ attempts to make sense of their experiences, creating a *double hermeneutic.* IPA typically draws on the accounts of a small number of people, with six participants considered sufficient (Reid et al. 2005). Therefore, using eidetic reduction via multiple readings and note taking, essential components were identified through an in-depth analysis of each transcript. The analysis focused on how respondents perceived their own individual experiences and, in this sense, the analysis was idiographic in nature, as opposed to attempting to conform narratives to a categorical system. Aided by discussions within the research team, notes were transformed into emergent themes, following which, relationships between narratives were established by searching for connections and clusters, so allowing the emergence of cluster themes to develop according to conceptual similarities.

## Results

The narratives were coded thematically, with the cluster theme of *identity and belonging* resulting from emergent themes relating to loss of group membership, identity threat, disconnection and childhood socialisation, vulnerability, and loss of belonging to family. The cluster theme of *depersonalisation* and *obedience* resulted from emerging themes of self-esteem, mental/physical health, negative self-image, ostracism, loss, shunning, suicide, rejection, loss of relationships and isolation. Themes were considered in relation to each other accounting for apparent differences which seemed to emerge as a function of “structural” influences on respondents’ narratives, as follows: (i) method of joining (born into the faith or conversion during adulthood), (ii) the exit method (disfellowshipped or voluntary exit) and (iii) retention of JW beliefs (retention of JW beliefs or their abandonment).

### Identity and Belonging

Respondents varied in the extent to which they said that they identified as JWs during their membership. Respondents who were disfellowshipped appeared to feel more strongly connected with the JWs prior to exit than those who chose voluntary disaffiliation. This was typified by accounts such as *“when I was about five…we became Jehovah’s Witnesses…so I felt there was a belonging, that was where I belonged yes”,* (Claire) and *“it was my tribe”* (Sue), indicating a visceral sense of connectedness to the JWs. Belonging appeared linked to personal identity, with these respondents, in particular, feeling that this had been shaped profoundly by their membership and their belief in the teachings as well as the wider JW culture and the social connections acquired through the organisation. Respondents commented that *“everyone I knew was a JW”* (Claire) and *“you are taught to have no friends outside the religion”* (Joanne). Level of identity was typified with expressions such as *“I was 101% JW”* (Sue). One interviewee, who disaffiliated voluntarily, expressed a divergent view and suggested that she had not felt a strong sense of belonging to the JWs during her membership, saying *“No, I never felt I belonged there”* (Jo), describing her previous self-identity in terms of her occupation (*“I was a nurse”)*. Overall, statements made by those who were disfellowshipped tended to indicate that this experience was conceived as more difficult than leaving voluntarily.

The extent to which identity and belonging were affected adversely during the transitioning out process seemed to be dependent upon the circumstances of leaving (how much control respondents felt they had) and on how long individuals had been socialised into the faith. Specifically, those who were disfellowshipped against their will appeared to find the leaving process more challenging than those who left voluntarily, as illustrated by Sue’s remark “*who was I?”* and *“who was I when I wasn’t a Jehovah’s Witness?*- *I was nobody…I felt invisible”.* Sue, who had been born into the religion indicated that without her JW identity, which was all she had ever known, she felt invisible and inconsequential. Similarly Claire, who was also socialised into the JWs from childhood describes a lack of belonging as *“you don’t have a belonging anymore…you feel lost again, it’s like being in a big ocean in a tiny boat, and no*-*one to rescue you…it’s a feeling hard to explain to anybody, unless they have actually been in that place”.* This description of feeling “lost at sea” with no rescue in sight evokes a sense of feeling forcibly cast adrift and isolated, severed from everything she had previously belonged to. Such statements from individuals who had been born and raised into the JWs indicate that early childhood socialisation may adversely impact post-exit identity transformation.

Considering the duration and extent to which individuals had been exposed to the faith allowed notable differences between interviewees to emerge. Those who had been born into the faith and socialised into it from infancy tended to report that they experienced more challenges to their identity and their sense of belonging than those who had joined the faith later in life. In this way, respondents in the former group talked about difficulties in finding a new identity and a new place to belong. Sue described her difficulty in how she viewed herself as *“It was very hard to interact with people because I didn’t know who I was…and I didn’t know how to do life as a non*-*JW…and I didn’t know what the boundaries were…I didn’t have any non*-*JW friends, so I only knew how to interact with my own kind*”. Claire also commented, *“you don’t really know who you are as a person, because you have been conditioned to think a certain way and to feel in a certain way, and so it very much affects how you feel about yourself…you don’t know yourself at all”*. An exception to this was John, who despite being born-into the faith, viewed his voluntary exit as an opportunity for him to discover his own identity, and to pursue his own life goals. Describing his membership as *“a cage of self*-*imprisonment”* which was *“very restrictive on your identity”,* he remarked that post-leaving *“That’s not what defines me, I am not anymore associated with the group. I have made my own choices, so I would say in terms of identity… I have been able to express my interests, views and thoughts more liberally and I think that’s a very good thing, especially as a young person”*. Here, it appeared that despite being born into the JWs, the positive effects of control (by leaving the JWs rather than being disfellowshipped) mitigated somewhat the effects of being shunned. Again, comments from respondents seem to indicate that being born into the JWs and subsequently disfellowshipped, among participants in this study, appears to be associated with more deleterious effects than being born into this religion and leaving it through choice.

Respondents who had converted to the JWs at a later stage during their life, on the other hand, tended to adjust more easily to life outside, in some cases returning to the life they had before they converted. Acknowledging that this was shaped by her Jewish upbringing, Sophie commented “*I think it depends whether or not you were indoctrinated in your formative years…my parents wanted me to be confident and outgoing, and were happy for me to be individual, and be a free thinker”.* Similarly, Jo, when asked if her sense of identity was affected when leaving the JWs, said *“No it wasn’t because…I was a senior nurse…I felt valued there”*. She further remarked that she made the decision to make new friends to create a new belonging *“I met Mark that night, as they say…one door closes and another opened…I was on my own but determined to make friends.*” Overall and with one notable exception, it appeared that the age respondents were introduced into the JWs had impacted significantly how they managed the shunning/ostracism they were experiencing. In respect to the overall theme of identity and belonging, the experiences of these two adult converts were different in that one was disfellowshipped, and one left through choice. However, it was evident that these respondents experienced less deleterious effects on their sense of identity than those raised as JWs, being more readily able to revert to their pre-JW identity, which is an impossibility for those born and raised JWs, and may help explain this finding.

Similarly, when reflecting on their experiences during the time of post-religious exit, it was apparent that respondents born-into the JWs who had experienced disfellowship had more difficulty in describing their identity at that time. Joanne described how it felt as *“you identify because you have friends, and you’ve been taught to have these friends and only these friends, who all have the same view and opinion that you have, and you don’t even allow yourself to envision yourself being friends with other people from outside your group…that’s your group and you belong in it”*. Sue described her identity as intrinsic to the JWs through the use of a metaphor: *“I do believe that when you’re brought up in it, it’s harder than if you have gone into it and then you leave. It’s different, it’s all in your hard wiring, the way you’re wired, everything was done as a JW.* Here, the permanence suggested by “hard wring” appears to add to the difficulties faced when trying to live life without the JW “lens”. The experience of leaving behind these social frames of reference was described as a disconcerting challenge for Joanne, who said *“even within the Jehovah’s Witnesses groups, there’s groups within that…there’s cliques within the Jehovah’s Witnesses group…I had my little clique, and then when it all went, I was on my own, completely on my own…I couldn’t identify…I was ostracised, kicked out of the group”*. Respondents also spoke about what seemed to be an “identity crisis” after their detachment from the faith indicating a need for them to construct a new identity outside of the JWs. *“I couldn’t measure myself…so as a Jehovah’s Witness you are constantly measuring yourself if you were good enough and about performance…you know…how many hours you did on the doors, how many times you answered up…and I no longer had this measuring stick…so…who was I?”* (Sue) It appeared that loss of social support and frames of reference outside of their JW environment caused a struggle to fit into the world they had been taught to avoid. Overall, being disfellowshipped appeared to hinder the pursuit of a new identity in the outside world especially among respondents who had been taught to restrict their friendships to only those within the JWs from childhood. While the process of leaving made interviewees experience grief following the severance of previous social connections, the formation of new (online) connections appeared to mitigate these adverse experiences to a degree. Respondents who indicated that they had gained support through social media described membership of groups as beneficial in two ways. First, they could “tell their stories” there, be understood, and find validation in their experiences with others who were able to relate to them. Second, new friendships could be forged with others who shared common experiences. It became apparent that a sense of support in providing a post-JW identity could be derived from membership of on-line support groups that had been set up by ex-JWs, as well as discovering a new sense of belonging. This was illustrated by expressions such as *“I have become involved with a group of people that have experienced some of the things I have experienced, I found that greatly helpful”* and*’being able to talk to other people who can relate to your experience…I can’t express enough how much that has helped me really”.* (Claire). In contrast, Sue explained that when she left the JWs there were no online groups to help adjustment “*there was no social*-*media so there was nobody to talk to, so that was extremely isolating and I think that was one of the hardest things…there wasn’t an ex*-*JW support group”.*

### Depersonalisation and Obedience

The theme of depersonalisation and obedience resulted from emergent themes relating to reduced self-esteem and well-being that respondents attributed to being disfellowshipped from the JWs. Although participants varied in the extent to which they described the extent to which their self-esteem and mental health were affected by their religious exit, this was perceived in terms of a loss of status within the JW community and their families. This was typified by expressions of personal identification with uncleanness and abhorrent acts such as “*it* [this religion] *has total control of your mind, it’s so damaging because it’s so entrenched in you…you are made to feel repugnant…you’re a dog returning to its vomit so how can you feel worthy in any way”* (Claire) and: *“it makes you feel like you’re dirty…like I was a really…bad…sinner…I did feel bad about myself”* (Joanne). Here, it seems that the derogatory way these respondents describe themselves was viewed to result from a failure to remain obedient to strict JW behavioural dogmas. Similarly, perceptions that their life course had been shaped by their long-term connection with the JWs, the wider JW community and the belief in the teachings was apparent with expressions such as “*I was disfellowshipped at 15…and the only way is to go back which involved sitting at the back of the kingdom hall and being ignored by everybody”* (Claire), and: *“I didn’t question anything, believed it 100%”* (Sue), and *“I still strongly believed it, I strongly believed in it and never stopped believing in it until just recent years, really”* (Joanne).It therefore appeared that these disfellowshipped JWs, who, as part of their socialisation into the JW faith had acquired a deep sense of belief, and felt an obligation to remain obedient to previous behavioural expectations that continued post-exit for quite some time.

The extent to which self-esteem was affected when exiting the JWs appeared to be mediated to some degree by the exit method and the passage of time. Diverse views were expressed as respondents described both short- and longer-term effects of being disfellowshipped. The immediate relief of no longer being held accountable for life choices was a desirable outcome for two respondents, as is illustrated by Joanne’s remark: *“seriously though, it was a relief, I did want that* [disfellowship] *at that point…I wanted to disassociate myself from it…I was under too much pressure…it was too hard”.* Claire expressed a similar reaction at the prospect of no longer having to be accountable for obedience to the high moral code of the JWs *“it was relief, I felt joy because I could go and do the things that previously were forbidden to me”.* These short-term effects appeared to evolve over time into feelings of loneliness and, what might be termed, an “unreality” as described by Joanne: *“I have always felt free since the day I got disfellowshipped…however other feelings that came into play, the feeling of being isolated…alone…with no support or friends…it’s like being in the film “I am Legend” it’s like the whole world has died, because they are your whole world, you don’t associate with anyone else…I was on my own in the world”.* It appeared that short-term consequences of exiting initially acted as a buffer against the effects of ostracism; however, over time, this seemed to break down and other longer-term effects emerged. Joanne’s narrative highlights how her social death and wider detachment from society interacted to contribute to these insidious consequences.

The data, in this way, indicate that for some interviewees longer-term effects such as self-condemnation and helplessness emerged after the passage of time. As Joanne remarked: *“your whole life you’re in fear of Armageddon and you’re going to die at Armageddon…the pressure was too much…I thought if I die at Armageddon I don’t care”.* This mental state, according to this interviewee, contributed to engaging in self-destructive behaviour: *“I nearly drank myself to death…I lost so much weight”.* Attributing this in part to the loss of the formerly close relationship she enjoyed with her mother before being disfellowshipped, Joanne noted that: “*My self*-*esteem took a massive hit, Mum doesn’t want anything to do with me…that has affected my self*-*esteem more than anything …. I felt like I had been in a car crash and I was very weak, and I couldn’t get up and I was on the floor”.* Here, longer-term psychological harms seemed to similarly arise from a sense of detachment from both the self and her family which the respondent felt had contributed to a lack of self-care and self-destructive behaviour. A similar picture of profound challenges faced shortly after leaving the JWs was painted by Claire, who described effects to her mental health as follows: *“It was a very very, VERY black time in my life because I literally had no*-*one…even my family were told to have no dealings with me…I was totally shunned by everyone”.* Other respondents also spoke about social death more metaphorically. Sue, for example, recalled, *“Overnight, it was like everybody that I knew in my life had got on a jumbo jet and it had gone down over the Atlantic … but there was no closure because everyone was still living their lives”.* Using a similar metaphor Joanne stated, “*imagine being on a plane, with all your family and friends, and the plane crashes…and only you survive, and all your family and friends are dead but you survived…but they’re not dead, they have dumped you…they are alive and well and getting on with their lives”*. Use of this metaphor highlights, how for these participants, the sudden loss of contact with loved was akin to grieving their living friends and family.

Other respondents, however, did not report any initial short-term feelings of relief after being disfellowshipped. Sue, for example described effects of leaving to her self-esteem as directly linked to her mental and physical health: *“I lost so much weight, my teeth looked too big for my face…I couldn’t eat…I wanted to die…I didn’t want to wake up in the morning…imagine every part of your body having a pin it in…that’s what it felt like…I wished I wasn’t breathing, because then I couldn’t feel the pain”.* Sue explained her declined mental well-being metaphorically and, echoing Joanne’s account above, describes how anxiety had impacted negatively her sense of self and had resulted in literal changes to her weight. Being raised and socialised into the JW religion emerged as a significant factor in how respondents dealt with life after leaving this religion. As Joanne commented: *“I was brought up in it, so I’d never known anything else…living in fear that Jehovah’s watching you…that Armageddon’s coming…and I totally believed it because you do… you believe what your mum tells you”.* It emerged that respondents who had been socialised into the faith early on felt more “conditioned” to feel and think in certain ways. This appeared to pose challenges to adjusting to post-JW life. As Claire comments: *“because you were conditioned to think in a certain way and to feel a certain way…you don’t have any self*-*worth really … everything I can think of, that has happened negatively in my life, I can pinpoint it to the time I was involved, from a child being erm, involved with Jehovah’s Witnesses”.* Commenting on how she carried on the JW legacy onto her own children, Claire described feeling guilty about raising her son in this religion which, she felt, was a contributory factor in his suicide after also being disfellowshipped: *“losing my son was indirectly caused by his involvement with Jehovah’s Witnesses, and his upbringing, and his being shunned”.* Here, the exit from the JWs was regarded a contributory factor in a suicide. When viewed in conjunction with the two attempted suicides by Sue, and the self-destructive behaviour reported by Joanne, the indication is that being disfellowshipped may potentially have serious implications to physical and mental health.

Further illustrating the impact of method of exit, one male respondent who had been raised in the JWs and had left through choice reported adjusting to post-JW life comparatively well : *“it did for a while* [affect self-esteem], *I think it does even now, but not negatively, again there are many ways your self*-*esteem can be impacted. I feel liberal, I feel free…I’ve been to two universities…I’ve met tons of people…joined committees…I’m quite an influential person now”* (John). This participant attributed this to having a sense of control over his situation which he felt was helped by having left with other family members and supporting each other throughout the exit process.

The apparent strong influence that childhood socialisation had on these former JWs was less apparent among those who had converted to the JWs as adults. Those who had converted as adults (Jo and Sophie) described their self-esteem as being generally intact after leaving the JWs, regardless of whether they had been disfellowshipped or not. For example, Jo who had been converted “on the doorstep” as an adult, and had left through her own choice 20, or so, years later described short-term lowered self-esteem: *“I suppose it did yes* [affect self-esteem], *I felt very down…I missed Jane, I grieved for her, it was as if she died, she was my best friend”.* In a similar way, Sophie downplays the impact of leaving on her self-esteem: *“I have never suffered with self*-*esteem issues, before I was a JW or after, or anything like that…I had it already…I wasn’t conditioned in my formative years, so I’ve never had a problem with self*-*esteem”.* At the same time, however, this respondent spoke about having experienced a mental breakdown after being disfellowshipped: *“I was disfellowshipped for exposing domestic violence, so it felt very unjust… it caused a lot of mental instability for me…I became so unstable I was hospitalised…I was told I was suffering with something called “reactive psychosis” because I was reacting to the injustice”.*

## Discussion

Building on previous research documenting how the process of exiting from high control religious communities may be associated with ostracism and feelings of abandonment (Friedson 2015; Holden 2002; Hookway and Habibis 2015; Lalich and McLaren 2010; Ransom et al. 2020; Testoni et al. 2019), this study examined how the leaving process impacted former members of Jehovah’s Witnesses. Analysis of the narratives yielded common themes documenting respondents’ sense-making through the process of “transitioning out”, which impacted adversely their sense of self, belonging, self-worth and mental well-being.

Participants in the current study were either disfellowshipped or had left of their own accord, and narratives appeared to indicate that the mode of exiting significantly impacted respondents’ sense of themselves in relation to others. In this way, respondents who had been disfellowshipped tended to report the experience of being “hard wired” to their JW identity to an extent. In contrast, respondents who had left on their own accord, tended to struggle less with establishing a new identity outside the faith, or with reverting to an identity more akin to their pre-JW life. By indicating that the mode of exit shapes identity transition, the current study contributes complementary insights to previous work which has tended to either examine solely the experiences of those who chose to leave the organisation (Testoni et al. 2019), or did not draw comparisons between those who may have had comparatively less agency in the leaving process (Lalich and McLaren 2010). In conjunction with these previous findings, the current research adds weight to the notion that people’s sense of agency may be an important influence on differential perceived threats to identity. Specifically, current findings suggest that adverse effects of felt religious ostracism may, to a degree, be tempered by a sense of control. Overall, the current study provides an initial qualitative indication that the experience of being disfellowshipped may pose a particularly strong threat to identity.

In contrast to findings of the current study indicating that a voluntary exit tended to be associated with more successful identity transition, Hookway and Habibis (2015) found that former JWs reported longer-term issues with adjusting their sense of self post-exit despite having chosen to leave the organisation. Similarly, although Lalich and McLaren (2010) did not draw explicit comparisons between JWs who chose to leave and those who were disfellowshipped, identity loss prevailed in the narratives. It is possible that differences in the samples between the current study and these previous investigations may help explain these disparities. Specifically, respondents in the Hookway and Habibis’ (2015) study were young people who had been socialised into the JWs from childhood and who had chosen to leave the JWs. In the Lalich and McLaren (2010) study, on the other hand, respondents were (mostly) disfellowshipped for homosexuality and, reporting a sense of self-hatred, faced the dual-task of needing to reformulate both their religious and their sexual identities. Seen in conjunction with current findings, it is therefore possible to posit that mode of exit interacts with wider influencing factors such as motivations for leaving and the extent to which respondents believe(d) the doctrine, and their identities are fused to that of the group (see Gomez, et al. 2011). Future research with larger sample sizes is clearly required to investigate in more detail the manifold influences on identity transition post-religious exit. Our findings suggest that a focus on the type of exit alongside wider social and structural influences (e.g. identity fusion, time socialised in religion and social support) could be worthwhile.

Findings from the study indicate further that respondents, whether disfellowshipped or not, experienced ostracism akin to a “social death” in respect to their family and friends. Those who were born into the JWs and were subsequently disfellowshipped seemed particularly likely to experience extreme and sudden losses which were sometimes described in metaphorical terms. Although those who had left voluntarily suffered similar losses, these respondents seemed to have anticipated ostracism to a greater extent and were more likely to come to terms with it through acceptance. As such, it appeared that leaving through choice mitigated the ill effects somewhat.

Identifying mode of exit as a potentially important moderator of adverse effects of religious exit and resultant ostracism, it becomes possible to identify parallels with the experiences of those whose loved ones are missing (Testoni et al. 2020). Testoni and her colleagues (2020) describe this as an “ambiguous loss”—whereby a person is definitively (and perhaps indefinitely) gone from individuals’ lives but may not be dead. While the sense of not knowing if someone is alive/dead is clearly different to experiences of former JWs, parallels can be identified. Both scenarios are characterised by emotional loss and a lack of closure preventing grief resolution. However, important distinctions must also be drawn. For example, the traditionally *shared* family experience of mourning and grieving, even ambiguously, a lost relative is not experienced by exJWs in this study, who, in some cases, had to deal with their experience of “grieving the living” alone.

Applying an identity reformulation theoretical lens to the current findings, it is also possible to pinpoint parallels between psychosocial processes underpinning transitioning out of high control religions and desistance from crime and substance abuse recovery. As Best and colleagues (2017) have argued, it seems that identity change comes about through a negotiated process of moving from existing social groups to new social groups, and finding a sense of meaning by negotiating a sense of self and belonging within these new groups (see also Best et al. 2016; Kay and Monaghan 2019). Although finding commonalities with the desistance literature in exploring the benefits of finding new social groups to support recovery, here too it is necessary to consider the differences, identified above in relation to ambiguous loss, with regard to the extent to which existing social support structures (e.g. of families) remain in place in social groups. Nevertheless, in support of previous work (Avance 2013; Best et al. 2018; Scharp and Beck 2017) the current findings suggest that forming new relationships is an important part of the process of identity transition after leaving the JWs, and may be associated with improved outcomes by mitigating effects of social losses. This is consistent with previous work indicating that the creation of new social bonds is intrinsically important to well-being following religious exit (Lalich and McLaren 2010; Nica 2019) and that engagement with online communities can facilitate this (Cheung and Lee 2010; Ridout et al. 2012). Findings from our small-scale study may therefore combine usefully previous investigations to serve as an early indication that applying theories of desistance and recovery to leaving high-cost religions may hold promise for future research and intervention efforts.

The need for more concerted efforts to better understand the effects of religious exit and how to support individuals’ post-faith transitioning (e.g. in online contexts; Nica 2019) are highlighted by consideration of the current findings in relation to mental well-being. In this way, respondents reported that leaving had impacted adversely their self-esteem and mental well-being which is consistent with previous research (Lalich and McLaren 2010; Ransom et al. 2020; Scheitle and Adamczyk 2010). In the current study, respondents’ data provide an early indication that having a sense agency in leaving the JWs may have lessened such adverse consequences. Similarly, it was apparent that those who left the JWs experienced a loss of belonging whereby they reported personal struggles with regard to finding a place in the world. This is consistent with the Friedson’s (2015) suggestion that JWs are taught from infancy that obedience to the organisation is crucial, and about the importance of rejecting common worldviews as threats to purity of spirituality. This notion may also help explain why former JWs in our study appeared to struggle with establishing a sense of belonging in a world which they, for a long period of time, had been taught to reject. More research is nevertheless required in order to unpick further how threats to mental-health and experiencing ostracism manifest over the longer-term, and the potential mediation of protective factors such as social support and method of leaving the faith.

Complimenting findings from other religions (Scheitle and Adamczyk 2010), the current study also found that the eschatology of the “doomsday” belief system associated with the JWs may have had a particular bearing on leavers’ mental well-being. As such, being cast out seemed, for some, to be associated with a sense of existential despair which appeared to be particularly apparent among respondents who had been born and socialised into the faith. This suggests that although all exit paths appear, to a greater or lesser extent, to be associated with experiences of religious ostracism, having a measure of control over the exit process may, to a degree, mediate deleterious effects to well-being. This aligns with previous JWs research (Lalich and McLaren 2010) and highlights that future investigations into exiting high control religions should be mindful of potential differences in the leaving process as a function of distinct belief systems.

## Conclusion and Limitations

A number of limitations need to be borne in mind when considering the findings of this small-scale qualitative study. First, while a small purposeful sample yielded detailed narratives and enabled in-depth consideration of these, it is unlikely that findings are representative or generalisable to the wider ex-JW community or indeed other religions. Interviewees’ narrative accounts are also likely to have been shaped by retrospective sense-making and could be subject to perceived demand characteristics, particularly as the interviewer is an ex-JW herself. As this is a cross-sectional study, the “snapshot” nature of the data should also be noted with caution, and further longitudinal qualitative and quantitative investigations are necessary.

Additionally, and by way of reflexive disclosure, it is noted that explanations and interpretations of the data may be coloured by the interviewer’s own experiences of leaving the JWs, outlined briefly above, as well as the prior research aims and theoretical stance rather than solely by the data collected. It is also possible that interpretations of the data may have been influenced by the views and personal experiences of the other members of the research team (one co-author was raised in a Christian tradition while the other was not raised in a religious environment) and the resultant interactions amongst the team as data and emergent themes were discussed.

In conclusion, this study sought to investigate qualitatively the experiences of former JWs to contribute a better understanding of how individuals “transition out” of the JWs and how this impacted their sense of who they are and their place in the world. Pointing to theoretical commonalities with the desistance and recovery literature, findings indicate that experiences of ostracism can be associated with diminished mental health, particularly among those socialised into the JWs from infancy and subsequently disfellowshipped. Findings also indicate that having a sense of agency and establishing new (online) social connections may help mitigate adverse consequences. Future research to unpick how these factors interact over time is warranted, as a better understanding of religious exits is likely to have implications for effective on- and off-line support provision.

## Data Availability

The data and materials for this study will be made available on request.
